# A High Number of Ring-Down Artefacts and an Irregular Pleural Surface Are More Commonly Observed in WHWTs Suffering from Idiopathic Pulmonary Fibrosis Compared to Control WHWTs

**DOI:** 10.3390/ani15202964

**Published:** 2025-10-13

**Authors:** Eugénie Soliveres, Emilie Pierrot, Aline Fastrès, Elodie Roels, Cécile Clercx, Géraldine Bolen

**Affiliations:** Department of Clinical Sciences, Companion Animals, Faculty of Veterinary Medicine, Fundamental and Applied Research for Animals & Health (FARAH), University of Liège, 4000 Liège, Belgium

**Keywords:** lung, dog, respiratory disease, ultrasound, pulmonary fibrosis, WHWT

## Abstract

**Simple Summary:**

Canine idiopathic pulmonary fibrosis is an interstitial lung disease reported in West Highland white terriers. Thoracic CT is part of the diagnosis of idiopathic fibrosis in human patients and dogs. However, some patients are presented for acute dyspnoea, preventing a CT scan from being performed under general anaesthesia. Thoracic ultrasound is used in human medicine as an adjunct tool for interstitial lung disease, including idiopathic pulmonary fibrosis. In veterinary medicine, thoracic B-mode US has been described for the diagnosis of various pulmonary diseases. The aim of this study was to describe the thoracic ultrasound findings in West Highland white terriers suffering from canine idiopathic pulmonary fibrosis, compared with presumably exempted West Highland white terriers. The presence of pulmonary lesions was higher in West Highland white terriers suffering from canine idiopathic pulmonary fibrosis compared to the presumably exempted group. Thoracic ultrasound is a non-invasive and well-tolerated tool that helps to differentiate West Highland white terriers with or without canine idiopathic pulmonary fibrosis.

**Abstract:**

Canine idiopathic pulmonary fibrosis (CIPF) is an interstitial lung disease reported in West Highland white terriers (WHWTs). B-mode ultrasonography (US) is used in human medicine as an adjunct tool for interstitial lung disease, including idiopathic pulmonary fibrosis. In veterinary medicine, thoracic US has been described as helpful for the diagnosis of various pulmonary diseases. The aim of this study was to describe the thoracic B-mode US findings in CIPF WHWTs, compared with those in control WHWTs. Twenty-seven WHWTs, including CIPF and control WHWTs, were prospectively enrolled. Standardised thoracic B-mode US was performed. The presence of an irregular pleural surface, ring-down artefact and peripheral nodules was assessed and scored for each location. An overall cumulative score was calculated by adding the individual scores of each location. WHWTs affected with CIPF had significantly higher overall scores compared to the control group. The ring-down artefact score was significantly higher in the CIPF group compared to the control group. No preferential location for the lesions was observed. A cut-off value of 15 ring-down artefacts for the entire thorax predicted CIPF in WHWTs with a sensitivity of 76.5% and a specificity of 80% (AUC 0.815). The present study describes B-mode US findings in CIPF WHWTs.

## 1. Introduction

Canine idiopathic pulmonary fibrosis (CIPF) of the West Highland white terrier (WHWT) breed is a chronic interstitial pulmonary disease that has common clinical and pathological features with idiopathic pulmonary fibrosis (IPF) and other interstitial lung diseases in humans [1,2,3]. Affected WHWTs are typically middle-aged to old [1,2]. Clinical signs include cough, exercise intolerance, laborious breathing, and/or cyanosis [1,3]. The definitive diagnosis is based on histopathology; however, pulmonary biopsies are not commonly performed in veterinary medicine because of the invasiveness of the procedure [1]. Furthermore, in dogs suffering from pulmonary fibrosis, the fragility of the lung parenchyma and the risks associated with anaesthesia are increased. In human medicine, the diagnosis of idiopathic pulmonary fibrosis is based on a set of converging factors, including clinical signs, anamnesis, and the appearance of lesions on chest CT scans. Lung biopsies are only performed after multidisciplinary discussion if other tests are inconclusive [4]. Similarly, in dogs, even though there is no standardised protocol, the diagnosis is based on signalment, compatible clinical signs and physical examination findings, diagnostic imaging, especially computed tomography (CT), and exclusion of other cardiorespiratory diseases [1,3,5]. Typical images on thoracic CT include a ground-glass opacity and a mosaic attenuation pattern [3,5]. Thoracic radiographs can be performed as a first step in the diagnostic approach but lack specificity [6]. Most dogs have an interstitial to broncho-interstitial lung pattern and, less frequently, patchy alveolar opacities with indistinct margins [7,8].

For some of these dogs, sedation or anaesthesia may be contraindicated, and a thoracic CT scan may not be performed or may not be available on site. Thoracic radiographs have a shorter acquisition time, but immobilisation in lateral recumbency might worsen respiratory distress for dogs presented with severe dyspnoea. Furthermore, radiographic images are nonspecific and do not rule out CIPF [6]. Ultrasonography (US) is a non-invasive, relatively inexpensive, and radiation-sparing technique that can be performed in the emergency room without transferring the patient. Thoracic ultrasonography is commonly used in veterinary medicine and human medicine as a rapid tool to diagnose pulmonary pathology [9,10,11]. In veterinary medicine, the use of thoracic ultrasound has increased during the last decade, with the development of point-of-care ultrasonography and focused assessment with sonography for trauma [9,10,11,12,13,14]. Its usefulness has been especially assessed for the detection of pulmonary cardiogenic oedema and pulmonary contusions [9,11]. In human medicine, thoracic ultrasound is also used in the diagnosis of idiopathic pulmonary fibrosis [15,16,17,18,19].

The aim of this study was to describe the thoracic B-mode point-of-care US findings in CIPF WHWTs, compared with those in control WHWTs.

## 2. Materials and Methods

The study design was prospective, observational, and case-controlled. Clinically CIPF-exempt (named control WHWT in the manuscript), middle-aged to old WHWTs and WHWTs with signs of CIPF were prospectively enrolled at the Veterinary Teaching Hospital of the University of Liège from March 2015 to May 2020 through the canine idiopathic pulmonary fibrosis project (http://www.caninepulmonaryfibrosis.ulg.ac.be and https://www.cvu.uliege.be/cms/c_7636211/fr/fibrose-pulmonaire-idiopathique-du-westie accessed on 5 June 2019). All procedures were approved by the Committee of Experimental Animals of the University of Liège, Belgium (permit number: 1649, date of approval: 27.01.2015, and permit number: 2245, date of approval: 05.04.2020) and performed with the signed consent of the owners.

WHWTs were considered to have CIPF according to compatible clinical signs (cough, exercise intolerance, respiratory distress, and/or crackles on lung auscultation) and thoracic CT findings. Compatible thoracic CT findings are the presence of ground-glass opacity and a mosaic attenuation pattern. Other less frequently associated findings are parenchymal and subpleural bands, consolidations, and nodules [3,5]. Eight of the included diseased WHWTs were thereafter confirmed to be affected with CIPF by post-mortem histopathological examination of lung tissue. Exclusion criteria were concomitant primary left or right cardiac disease associated with signs of cardiac failure. WHWTs were classified as exempt from CIPF if they had no history of pulmonary or cardiac disease, if no primary cardiac disease with signs of cardiac failure was diagnosed on echocardiography, and if physical examination was within normal limits. For each dog, complete blood work, including arterial blood gas, a complete echocardiographic examination, and endoscopy with bronchoalveolar lavage, as well as a 6 min walking test, was performed.

### 2.1. Thoracic Ultrasonography

Three different ultrasound machines were used: the first machine with a 4 to 13 MHz linear and a 4 to 12 MHz curvilinear transducer (MyLab CalssC, Esaote, Maastricht, The Netherlands), the second machine with a 7.5 to 10 MHz linear and a 5 to 7.5 microconvex MHz transducer (Prosound alpha 10, Aloka, Tokyo, Japan) and the third machine with a 5 to 18 MHz linear and a 4 to 10 MHz microconvex transducer (Arietta 850 SE, Hitachi, Tokyo, Japan) from 2015 to 2020. The ultrasound probe and frequency were adapted according to the dog’s conformation, as in daily clinical use, with a preference for the linear probe when penetration was sufficient.

Thoracic B-mode ultrasonography was performed in sternal recumbency or standing position, depending on the dog’s preference. Hair was not clipped. Alcohol with or without gel was used as a coupling agent. Four intercostal spaces were evaluated in a transverse orientation (parallel to the ribs) on the right and left sides. The first location evaluated the cranial lobes, and the probe was placed at the level of the 2nd or 3rd intercostal spaces at the level of the ventral half of the thorax. The 2nd location evaluated the right middle lung lobe and the caudal part of the left cranial lung lobe, and the probe was placed at the level of the 4th or 5th intercostal spaces at the ventral half of the thorax. The third location evaluated the dorsal mid-part of the thorax, and the probe was placed at the level of the 6th or 7th intercostal placed at the level of the junction between the ventral half and the dorsal half of the thorax. Finally, the 4th location evaluated was the caudal lung lobes, and the probe was placed at the level of the 7th, 8th, or 9th intercostal spaces in the more lateral area of the thorax at the level of the dorsal half of the thorax. For each location, the selected intercostal space was the one allowing the best image quality and was adapted for each patient.

**Figure 1:**
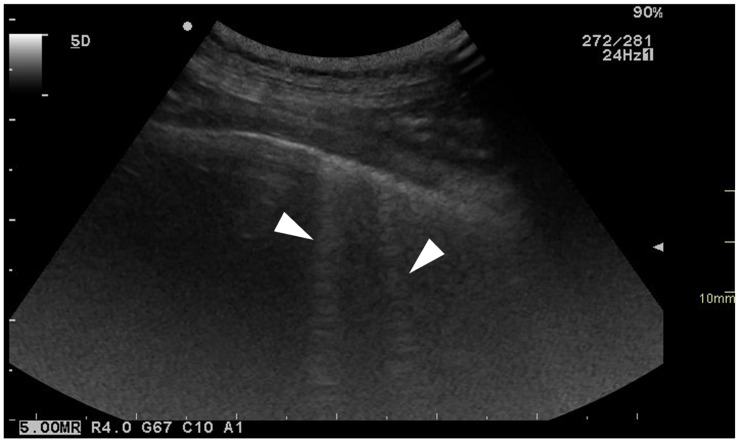
Ultrasonographic image of the thorax of a WHWT affected with CIPF showing ring-down artefacts (white arrows). The image was obtained in a transverse orientation (parallel to the ribs).

**Figure 2:**
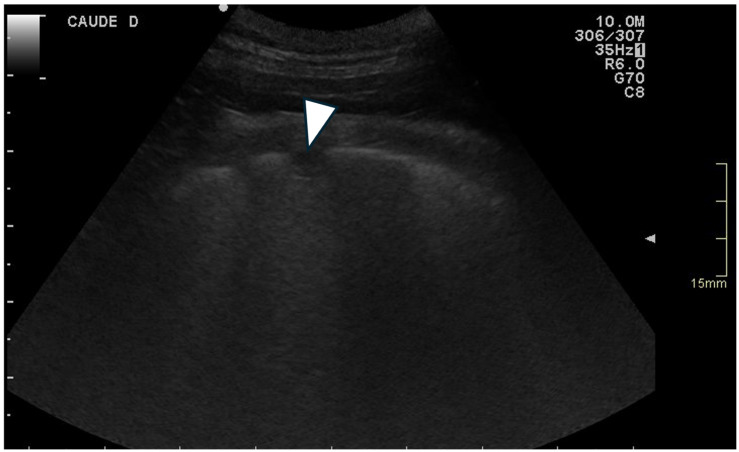
Ultrasonographic image of the thorax of a WHWT affected with CIPF showing a peripheral hypoechoic pulmonary nodule (white arrow). The image was obtained in a transverse orientation (parallel to the ribs).

**Figure 3:**
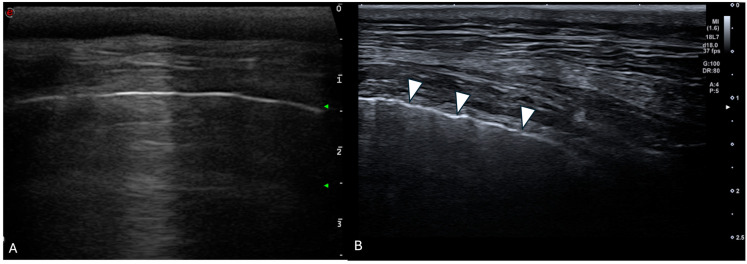
Ultrasonographic image of the thorax of a control WHWT (**A**) and a WHWT affected with CIPF (**B**). (**A**): smooth pleural surface, (**B**): irregular pleural surface (white arrows). The images were obtained in a transverse orientation (parallel to the ribs).

For each location, the presence and the number of ring-down artefacts (Figure 1) were counted. Depending on the number of artefacts, a score was obtained from 0 to 3 (Table 1). A similar method was applied for other lung lesions, such as peripheral hypoechoic pulmonary nodules or hypoechoic pulmonary areas (Table 1, Figure 2). The pleural surface was defined as smooth or irregular (Table 1, Figure 3)—scores of 0 or 1—and pleural sliding was assessed as present or absent (Table 1)—scores of 0 or 1. Pleural effusion was assessed as present or absent—scores 0 or 1 (Table 1). The scores were summed for each location and each side, and a total score out of 72 was attributed to each dog. Examinations were performed by the same operator (GB) on all dogs in a blinded fashion.

The overall lung ultrasound score was assessed for all included WHWTs and corresponded to the sum of the scores of each lesion in each location. The median number of ring-down artefacts and pulmonary lesions was scored per location on each side and summed for the whole lung. They were compared between CIPF and control WHWTs.

### 2.2. Statistical Analysis

Statistical analyses were performed using commercially available software (Excel, Microsoft Office, and XLStat software, version 2021.4; Addinsoft SARL, International). A Shapiro–Wilk test was applied to test the normality of the distribution of continuous variables. Comparisons between groups were performed using a Mann–Whitney test. A receiver operator characteristic (ROC) curve analysis was generated to determine the optimal cut-off value of the total number of ring-down artefacts for the prediction of CIPF in WHWTs. Proportions were compared by a chi-squared test. Statistical significance was set at *p* < 0.05.

## 3. Results

### 3.1. Dogs

Twenty-seven WHWTs were enrolled: seventeen WHWTs affected with CIPF and ten presumed exempt WHWTs. There was no significant difference in age or sex between groups. WHWTs affected with CIPF weighed significantly more than control dogs (median = 9.9 kg and median = 8.25, respectively, *p* = 0.03).

Concomitant comorbidities in the control group included an adrenal mass in one dog, atopy in one dog, kerato-conjunctivitis sicca and a palpebral mass in one dog, chronic diarrhoea and urinary incontinence in one dog and chronic rhinitis in one dog. Concomitant comorbidities in the CIPF group included Cushing’s disease, kerato-conjunctivitis sicca and a circumanaloma in one dog, bilateral suppurative otitis in one dog, Cushing’s disease and diabetes mellitus in one dog, severe gingivostomatitis in one dog, chronic bronchitis in one dog, kerato-conjunctivitis sicca and atopy in one dog, atopy in one dog, and atopy, facial paralysis and a removed mammary mass in one dog.

Fourteen dogs have died since the end of the study, including one control WHWT. The date of death ranged from 1 day to 4 years after the date of the ultrasound exam. The thirteen other patients were lost to follow-up. Histopathological analysis was performed in eight of the CIPF WHWTs and was consistent with pulmonary fibrosis. None of the control dogs developed clinical signs compatible with pulmonary fibrosis.

### 3.2. Clinical Data

In WHWTs affected with CIPF, the mean duration of clinical signs was 3 months (range: 2–24 months). The most reported clinical signs were cough in 12 dogs and exercise intolerance in 11 dogs. Respiratory distress was present in eight dogs during clinical examination, and cyanosis in five dogs. Crackles were heard during auscultation in 14 dogs.

Bronchoscopy was performed in 14 dogs, including 10 CIPF dogs and 4 control dogs, and revealed grade 1 tracheal collapse in 2 CIPF dogs and 2 control dogs and grade 2 tracheal collapse in 3 CIPF dogs (*p* = 0.350), bronchomalacia in 7 CIPF dogs and 2 control dogs (*p* = 0.480), and mucus in the bronchi in 5 CIPF dogs and one control (*p* = 0.393).

Echocardiography was normal in 12 dogs, including 7 control dogs. Mild to moderate pulmonary hypertension was diagnosed in eight dogs (including three control dogs), and moderate to severe pulmonary hypertension was diagnosed in six CIPF dogs. One CIPF dog had a stage B1 myxomatous mitral valve disease without evidence of left ventricular systolic dysfunction.

At the time of diagnosis, eight WHWTs affected with CIPF were under therapy. Treatments in CIPF group were theophylline (dose ranging from 8.85 to 11 mg/kg BID) in three dogs, sildenafil (1 mg/kg BID) in two dogs, and pimobendane (0.38 mg/kg), codeine (0.9 mg/kg BID), prednisolone (1 mg/kg BID), benazepril (0.43 mg/kg SID) and spironolactone (3.51 mg/kg SID), fenspiride (2 mg/kg BID), acetylcysteine (10 mg/kg TID), and fluticasone (one puff BID) in one dog each. One dog received theophylline at an unknown dosage, and another dog received ursodeoxycholic acid, trilostane, and insulin. No control dog was under therapy at the time of the study. Sildenafil was initiated in two CIPF dogs because of previously diagnosed pulmonary hypertension. In one of these dogs, pimobendan had already been initiated by the primary veterinarian. At the time of the echocardiography, no left ventricular systolic dysfunction was observed. Benazepril and spironolactone were initiated in one CIPF dog by the primary veterinarian before echocardiography and were discontinued after the echocardiography.

### 3.3. Thoracic Ultrasound Results

#### 3.3.1. Transducers

Linear transducers were used in nine dogs (seven control and two CIPF-affected WHWTs), and curvilinear transducers were used in eighteen dogs (three control and fifteen CIPF-affected WHWTs). Curvilinear transducers were more often used in WHWTs affected with CIPF compared with control dogs (*p* = 0.0019). No significant difference was observed between the median weight of dogs scanned with linear transducers or with curvilinear transducers (9.5 and 9.45 kg, respectively, *p* = 0.41).

The procedure was well-tolerated and entirely performed in all patients included in the study.

#### 3.3.2. Total Score and Repartition of the Lesions

WHWTs affected with CIPF had a significantly higher overall score compared to the control group (median = 18, range = 9–36 vs. median = 10, range = 1–28, respectively, *p* = 0.005).

No pleural effusion and no signs of reduced pulmonary sliding were observed in any of the dogs.

In the CIPF group, the higher-total-scored lesions were ring-down artefacts (median = 9, range 1–18), followed by irregular pleural surface (median = 7, range 0–8) (*p* = 0.017). A summary of the maximum score measured in CIPF-affected and control WHWTs for each lesion is displayed in Table 2.

#### 3.3.3. Ring-Down Artefacts

**Figure 4:**
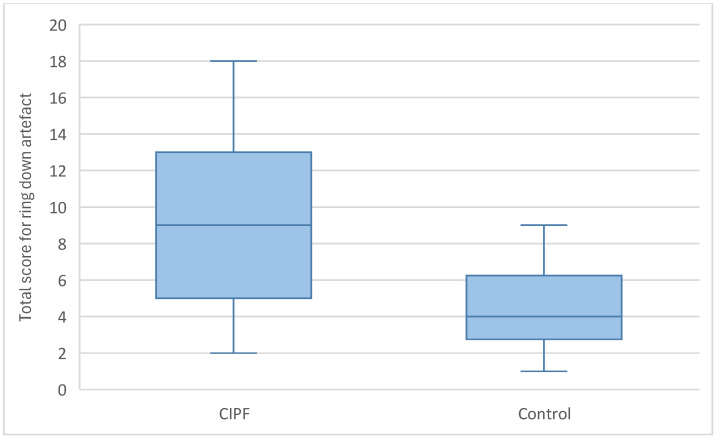
Comparison of the total ring-down artefact scores between CIPF WHWTs and control CIPF.

**Figure 5:**
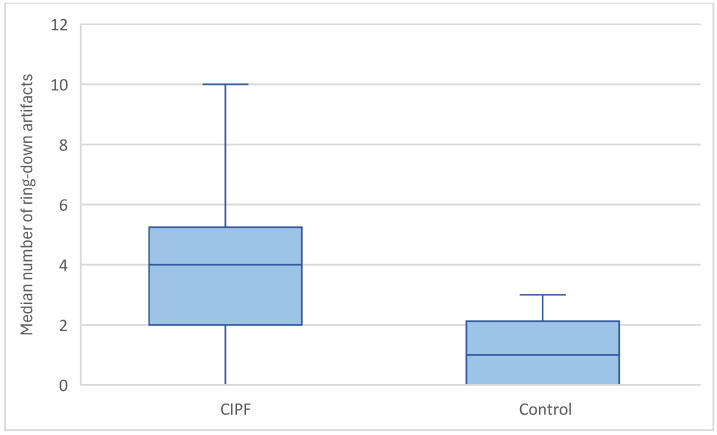
Comparison of the higher median number of ring-down artefacts per location between CIPF WHWTs and control CIPF.

Ring-down artefacts were observed in at least one location in all dogs included in the study. The total score for ring-down artefacts was significantly higher in WHWTs affected with CIPF (median = 9, range = 2–18) compared with controls (median = 4, range = 1–9) (*p* = 0.004) (Figure 4). WHWTs affected with CIPF had a higher median number of ring-down artefacts per location compared with control dogs (median = 4, range = 0–10 vs. median = 1, range = 0–3, respectively, *p* = 0.003) (Figure 5). No dog included in the control group had a score of 3 per location regarding ring-down artefacts. No preferential location for the lesions was observed. A cut-off value of 15 ring-down artefacts in the entire thorax discriminated WHWTs affected with CIPF from controls with a sensitivity of 76.5% and specificity of 80%, and an area under the receiver–operating characteristic curve (AUC) of 0.815.

#### 3.3.4. Pleural Surface

An irregular pleural surface was observed in 16 of the 17 WHWTs affected with CIPF and in 8 of the 10 control dogs (*p* = 0.26). No significant difference was observed regarding total score for irregular pleural surface between groups (median = 7, range = 0–8 in CIPF vs. median = 4.5, range = 0–8 in controls) (*p* = 0.057). There was no significant difference between groups regarding the median score for pleural surface irregularity per location (median = 1, range = 0–1, vs. median = 0.75, range = 0–1, respectively, *p* = 0.324). No preferential location for the lesions was observed.

#### 3.3.5. Other Lesions

Other lesions, such as peripheral hypoechoic nodules or hypoechoic areas, were present in 13 of the 17 CIPF dogs and in 3 of the 10 control dogs (*p* = 0.018).

The total score for other lesions was significantly higher in WHWTs affected with CIPF (median = 4, range = 0–18) compared with controls (median = 0, range = 0–14) (*p* = 0.024). No preferential location for the lesions was observed.

## 4. Discussion

This study highlights that thoracic ultrasound is a useful, non-invasive and well-tolerated tool that could help in the clinical assessment of WHWT affected with CIPF. The presence of a significantly higher number of ring-down artefacts, distributed across all intercostal spaces, was a consistent finding in affected dogs. Using a cut-off value of more than 15 ring-down artefacts across the thorax, ultrasound showed good diagnostic accuracy in distinguishing WHWTs with CIPF from unaffected controls. These findings are consistent with the diffuse and bilateral nature of CIPF and suggest that thoracic ultrasound may be used to support a clinical suspicion of the disease in practice.

During ultrasonographic evaluation of normal lungs, only the surface of the lungs is visible [20]. Because lungs are highly reflective, they appear as an echoic linear structure with distal multiple, horizontal and parallel reverberation echo artefacts representing multiple reflections of the pleural line [20]. This artefact avoids visualisation of the deeper pulmonary structures [20]. The parietal pleura is not always visualised, and when it is, it appears as a thin and smooth hyperechoic line that can be distinguished from the visceral pleura because of the gliding of the lung during respiration [21]. Reverberation artefacts include ring-down artefacts, sometimes called B-lines, and comet tail artefacts.

Ring-down artefacts are caused by the impedance gradient between fluid/tissue and surrounding air and occur when the transmitted ultrasound energy causes resonant vibrations within fluid trapped between a tetrahedron of air bubbles [20,21]. These vibrations create a continuous sound wave that is transmitted back to the transducer [20]. Consequently, ring-down artefacts may be observed in any alveolar or interstitial pulmonary pathology and are not specific to a disease [22,23,24]. Ring-down artefacts have been described in people and horses with atelectasis, pulmonary fibrosis, acute respiratory distress syndrome, pulmonary haemorrhages, pneumonia, or lung cancer [10,25,26,27].

Several studies have described the usefulness of thoracic ultrasonography for the diagnosis of acute pulmonary disease in dogs and cats [12], especially in the case of cardiogenic pulmonary oedema [9,28,29,30]. Similar to WHWTs suffering from CIPF, crackles may also be heard during pulmonary auscultation in dogs with pulmonary cardiogenic oedema. Patients with cardiogenic pulmonary oedema had a significantly higher number of sites with ring-down artefacts compared to patients with non-cardiac disease [9]. Cardiogenic pulmonary oedema can be suspected when two or more positive sites are present [31] or when more than four ring-down artefacts are visible on the pericardial lung ultrasound [28]. A positive site is defined as three or more ring-down artefacts [31]. However, a number of three or more ring-down artefacts in a single intercostal space had been found to indicate alveolar-interstitial syndrome in dogs [32]. A similar trend was observed in our study, where the median number of ring-down artefacts per intercostal space was four in WHWTs affected with CIPF compared with age-matched control dogs, who had a median number of one ring-down artefact per intercostal space. Because echocardiography was systematically performed for inclusion in the CIPF study project to rule out concomitant primary left cardiac disease associated with left cardiac failure or primary right cardiac disease and to assess the presence or not of pulmonary hypertension, the cause of ring-down artefacts cannot be attributed to cardiogenic pulmonary oedema in our cohort of dogs. For application in routine clinical practice, even in the absence of a board-certified cardiologist, basic echocardiographic evaluation (such as the LA/Ao ratio) performed alongside the lung exam can help exclude left-sided cardiac failure, which is crucial for interpreting ultrasound findings in the context of interstitial disease [33,34].

The location of ring-down artefacts might help to diagnose the cause of respiratory distress [22]. Extrapolated to the radiographic location of pulmonary diseases, the location of ring-down artefacts might be suspected to be cranio-ventral in case of bronchopneumonia, peri-hilar and caudo-dorsal in case of cardiogenic oedema, and without preferential location in case of pulmonary contusions [35]. Nonetheless, ring-down artefacts in the case of cardiogenic pulmonary oedema have a multifocal location, with the right and left middle sites more commonly affected [9,22]. The authors explained this discrepancy between thoracic ultrasound and thoracic radiography because pulmonary oedema did not extend to the periphery of the caudodorsal lungs [9]. In the case of bronchopneumonia, the right middle site alone is more commonly affected [9,22,36]. In our study, no preferential location was observed, and ring-down artefacts were present in all intercostal spaces, including caudo-dorsal sites. This difference can be explained by the fact that, as previously described, CIPF disease affects the entire lungs without a preferential location [3,5]. Ring-down artefacts can consequently be observed in the periphery of the lungs in the case of CIPF. Pulmonary contusions can have a random distribution and could be confused with CIPF; however, the history can help distinguish pulmonary contusions from CIPF.

In humans, the diagnosis of IPF is based on a multidisciplinary approach [4,37,38]. A typical usual interstitial pneumonia (UIP) pattern on CT, combined with compatible clinical signs, is sufficient to diagnose IPF [4,37,38]. UIP pattern consists of honeycombing lesions typically located in the dorsal, basal, and subpleural regions of the lungs with or without bronchiectasis in the absence of findings that suggest a diagnosis other than IPF [4,37,38]. Thoracic ultrasonography is an accurate tool for the diagnosis of pneumonia, pneumothorax, acute pulmonary oedema, and pleural effusion in humans [39]. It has been found to be highly sensitive and specific for the diagnosis of lung diseases [40], especially for diagnosing pulmonary interstitial disease (ILDs) [39,40,41,42]. Ring-down artefact is considered the hallmark of diagnosing ILDs in human patients [39]; they are observed in higher numbers in patients with ILDs compared to patients without ILDs [41]. ILDs were defined as the presence of three or more ring-down artefacts per intercostal space in a longitudinal approach, with a distance of no more than 7 mm between two lines [17]. The typical ultrasonographic appearance of the lung in the case of IPF has been described as a regular or irregular thickening of the hyperechoic pleural line, an irregular pleural line, an increased number of ring-down artefacts (more than three), and evidence of subpleural nodulations [17,18,19,42]. Consequently, the presence of ring-down artefacts cannot be considered as an indicator of lung fibrosis when observed alone; other findings are needed [17]. Furthermore, in another study performed on human patients with IPF, thickening of the parietal pleura was the only finding observed in patients with a mild form of IPF [17]. In the same study, the absence of pulmonary sliding was observed only in severe cases. Another study observed ring-down artefacts in 80% of patients suffering from IPF, pleural line irregularity in 78%, pleural line thickening in 56%, subpleural changes in 44% and decreased lung sliding in 44% [42]. Regarding ring-down artefacts, pleural line irregularity and subpleural changes, the same trend was observed in our study in canine patients suffering from CIPF, because they were present in 100%, 94% and 58%, respectively. No decreased lung sliding was observed in our cohort of dogs. These differences could be attributable to differences in the physiopathology of pulmonary fibrosis in dogs and humans or because of a less severe remodelling of the lung architecture in canine patients [43,44].

In IPF patients, the median number of ring-down artefacts, the distance between two adjacent ring-down artefacts, and the average thickness of the pleural line obtained by ultrasonography had a positive and significant correlation with the CT score [18,45]. Distance between ring-down artefacts was not measured in this study but could be a marker of disease severity, such as in human medicine. In the same way, the distance between two ring-down artefacts, the irregularity of pleural lines, the thickness of pleural lines, and the absence of lung sliding were associated with the severity of restrictive pulmonary functions [45].

Finally, the last finding in our study was subpleural lesions. In human patients affected with IPF, subpleural nodules observed on thoracic ultrasound have not been associated with the presence of a nodular pattern or of a more severe honeycombing pattern on thoracic CT [19]. As for the other ultrasonographic findings, subpleural lesions are not a specific finding in the case of pulmonary fibrosis [19]. In our study, subpleural lesions were observed in a significantly higher number of CIPF WHWTs compared to the control group. However, similarly to human patients, the causes of subpleural lesions in dogs are various. Subpleural nodules can be observed in various pathological conditions, including neoplastic or infectious diseases in dogs, such as fungal pneumonia, bacterial pneumonia, bronchitis, dynamic airway collapse, and congenital pulmonary anomalies [31]. In particular, a study found a high specificity and sensitivity for lung ultrasound in the diagnosis of angiostrongylosis in young dogs with respiratory distress, the typical finding being subpleural nodules [46]. In this study, the main reported ultrasound lesions were multiple subpleural pulmonary nodules associated with concomitant ring-down artefacts. The lesions were systematically bilateral and affected mainly the caudodorsal areas. They were not associated with pleural irregularity, contrary to CIPF dogs of the present study. These results differ from our study, where the lesions were mainly ring-down artefacts and no preferential location was observed.

In contrast to thoracic radiographies, thoracic ultrasound is a non-ionising technique and can be performed at the bedside of the patient [9,12,47,48]. In human patients, thoracic ultrasound can help in selecting patients who need CT for diagnosis or as a triage tool in clinics without a CT scanner [42]. Furthermore, in the case of connective tissue disease, a type of interstitial disease, thoracic ultrasound may be a useful tool at the time of diagnosis and for follow-up [49]. In the same way, thoracic ultrasound can be used in dogs to help discriminate between dogs with suspicion of CIPF and dogs with other causes of acute respiratory distress and guide toward thoracic CT if necessary.

Our study has several limitations. The first limitation is that a definitive diagnosis was not available in nine dogs of the CIPF group, because the dogs are still alive or because post-mortem biopsies were refused by the dogs’ owners. Because of the invasiveness of the procedure, no pulmonary biopsies were performed during the study, and the diagnosis was only based on clinical presentation, clinical examination, and results of thoracic CT. Because of the insidious nature of the disease, some dogs categorised as non-clinical may have been at an early stage of the disease. However, to our knowledge, no dogs included in the control group developed clinical signs compatible with CIPF.

Different machines and different probes were used during our study. The linear probe was used first, but if the patient was overweight or if the beam attenuation was excessive, curvilinear probes were used, as in daily clinical use. However, the number of ring-down artefacts can vary depending on the probe or the total gain. A high-frequency linear probe reduces the number of artefacts, and a low-frequency convex probe increases the number of these artefacts [50,51,52]. Similarly, an excessive total gain is associated with a higher number of ring-down artefacts [50,52]. In our study, WHWTs affected with CIPF weighed significantly more than control dogs, and micro-convex probes were more often used in WHWTs affected with CIPF compared with control dogs. The use of different probes, especially micro-convex probes, could have falsely increased the number of ring-down artefacts; however, a veterinary study has found that curvilinear and linear probes can be used interchangeably and that the number of ring-down artefacts was not significantly different between these two probes [53].

Another limitation is the small number of dogs included in each group, which could increase the risk of type II statistical error. Further studies with a higher number of dogs should be considered in the future to confirm our result or to assess the usefulness of thoracic ultrasound for the follow-up and progression of CIPF. However, because of the relatively low prevalence of CIPF, it may be difficult to recruit WHWTs with CIPF.

Finally, this study only focused on CIPF disease. It would have been interesting to compare CIPF disease with other pulmonary diseases. Furthermore, CIPF WHWTs have frequent comorbidities such as bronchitis or bronchomalacia, which could have led to pulmonary lesions. However, control WHWTs were also affected by other pulmonary diseases.

## 5. Conclusions

The presence of a high number of ring-down artefacts and an irregular pleural surface is observed in WHWTs affected with CIPF compared to unaffected WHWTs. The presence of three ring-down artefacts per location and more than sixteen ring-down artefacts in the entire thorax could help distinguish WHWTs affected with CIPF from unaffected WHWTs. Generalised distribution of ring-down artefacts and an irregular pleural surface could help in the diagnosis of interstitial lung diseases in dogs. However, the presence of ring-down artefacts is not specific and there are overlapping exits between the location and the number of ring-down artefacts depending on the lung disease. History and a comprehensive thoracic ultrasound, including a basic cardiac assessment, can aid in ruling out alternative causes such as cardiogenic oedema and should be part of the diagnostic workflow in suspected CIPF cases. Due to several limitations of the study, these results should be considered preliminary. Further studies are warranted to assess the ultrasound images in comparison with the severity of the CT images and to evaluate the usefulness of the thoracic ultrasound in the follow-up of affected patients. Comparison with other lung diseases may also be considered to help differentiate lesions caused by CIPF from those caused by other lung diseases.

## Figures and Tables

**Table 1:** Grading of the different pulmonary lesions observed on thoracic B-mode point-of-interest ultrasonography.

	Ring-Down Artefacts	Lung Lesions	Pleural Surface	Pleural Sliding	Pleural Effusion
Score 0	0	0	Smooth	Present	Absent
Score 1	1–5	Small hypoechoic nodules	Irregular	Absent	Present
Score 2	6–10	Larger hypoechoic areas			
Score 3	>10	Both			

**Table 2:** Maximum score measured in control and affected WHWTs for each lesion.

	Score	Ring-Down Artefacts (Dogs = n)	Lung Lesions (Dogs = n)	Pleural Surface (Dogs = n)	Pleural Sliding (Dogs = n)	Pleural Effusion (Dogs = n)
Control dogs	0	0	7	2	10	10
	1	8	1	8	0	0
	2	2	1			
	3	0	1			
CIPF dogs	0	0	4	1	17	17
	1	5	2	16	0	0
	2	9	10			
	3	3	1			

## Data Availability

The raw data supporting the conclusions of this article will be made available by the authors on request.

## Abbreviations

The following abbreviations are used in this manuscript:

CIPF	Canine idiopathic pulmonary fibrosis
WHWT	West Highland white terrier
IPF	Idiopathic pulmonary fibrosis
CT	Computed tomography
US	Ultrasonography
UIP	Usual interstitial pneumonia
ILDs	Pulmonary interstitial disease
